# Analysis of definitive chemo-radiation outcomes in anal cancer: insights from a tertiary cancer center in the MENA Region

**DOI:** 10.3389/fonc.2023.1333558

**Published:** 2024-01-04

**Authors:** Tala Alawabdeh, Ramiz Abuhijlih, Issa Mohamed, Saif Alnasraween, Hazem Ababneh, Reem Turfa, Sanad Alsunna, Yacoub Khzouz, Fawzi Abuhijla

**Affiliations:** ^1^ Department of Medical Oncology, King Hussein Cancer Center, Amman, Jordan; ^2^ Department of Radiation Oncology, King Hussein Cancer Center, Amman, Jordan; ^3^ Department of Internal Medicine, University of Jordan School of Medicine, Amman, Jordan; ^4^ Department of Radiation Oncology, Massachusetts General Hospital, Boston, MA, United States; ^5^ Department of Pathology, King Hussein Cancer Center, Amman, Jordan

**Keywords:** anal cancer, chemoradiation, hemorrhoidectomy, MENA (Middle East and North Africa), overall survival

## Abstract

**Background:**

Outcomes of chemo-radiation (CRT) for anal cancer in Middle East and North Africa (MENA) are scarce. We aim to report treatment outcomes for anal cancer treated at tertiary cancer center, with a particular focus on patients managed with non-oncological surgery prior definitive CRT.

**Methods:**

We conducted a retrospective review of patients diagnosed with locally advanced anal carcinoma, who underwent definitive CRT King Hussein Cancer Center, from January 2007 till January 2020. Patient demographics and disease characteristics were extracted, and a univariate chi-squared test was employed to assess the impact of chemotherapy type, HPV status, and pre-treatment non-oncological surgery on outcomes, including complete remission (CR), disease-free survival (DFS), and overall survival (OS). Kaplan–Meier tests were employed to analyze the obtained survival data.

**Results:**

Among the 34 initially identified patients, 30 were eligible, 24 (80%) achieved CR. Notably, 20 out of 21 HPV positive patients achieved CR, versus 1 out 4 HPV-negative achieved CR*, p*=0.006The 5-years OS for HPV-positive patients was 89% compared with 25% for HPV-negative, *p*=0001. There was no statistical significant difference in patients outcomes as regard type of chemotherapy, radiation technique and non-oncologic resection prior to CRT.

**Conclusion:**

Herein, we reported the first series of anal cancer from our region. CRT had yielded an oncologic outcome comparable with series in the literature. HPV-positive patients demonstrated better results. Moreover, we found non-oncologic resection prior to CRT did not seem to impact the outcomes. Further studies are warranted to overcome the limitations of our study.

## Introduction

Anal cancer is a rare malignancy of the gastrointestinal (GI) tract, comprising approximately 1-2% of all GI cancers. The predominant type is squamous cell carcinoma (SCC), with a higher incidence observed in individuals aged 55-64 years. Additionally, it is more prevalent in female gender (1).

The incidence of SCC has remained steady in certain Asian countries such as India, Japan, and Singapore. An analysis, covering the years 1989 to 2007, demonstrated a relatively stable number of new cases over that period (2). On the other hand, the incidence of anal carcinoma in Middle East and North Africa (MENA) remains lower than other regions in the world (3).

In Jordan, anal cancer constitutes 0.2% of all newly diagnosed malignancies, as per the national cancer registry 2016 (4), which is lower than the global incidence of 0.5%, according to Surveillance, Epidemiology, and End Results (SEER) data (5).

Human papillomavirus (HPV) is the primary risk factor for anal cancer. Other contributing factors include immune deficiency which is linked to Human Immunodeficiency Virus (HIV) infection and immunosuppressiv medications, as well as sexually transmitted diseases, chronic inflammation, fistulas, and tobacco use (6).

A growing body of evidence suggests that oncogenic strains of HPV, particularly subtypes 16 and 18, play a role in the development of SCC in various anatomical sites beyond the anal canal, including cervical and head and neck cancers. In a study conducted by Frisch and colleagues, HPV DNA was identified in 88% of patients with anal cancer, HPV subtype 16 was implicated in 73% of the cases of anal cancer (7).

HPV infection has been linked to sexual practice. Which includes having first intercourse at earlier age, a greater number of male sexual partners, and engaging in receptive anal intercourse (8). These practices have increased in recent years, especially in high-income countries (9). This may explain why the incidence in Asia and MENA region is lower (10).

Hemorrhoids are not widely acknowledged as a risk factor for anal cancer. Nevertheless, persistent irritation associated with long-term hemorrhoids could potentially lead to the development of squamous cell carcinomas. Consequently, it is advisable to conduct histopathologic examinations of hemorrhoidectomies specimens (11). Moreover, anal carcinom usually presents with rectal bleeding and sensation of a mass, which mimic symptoms associated with benign anal conditions like perianal hemorrhoids and fissures. This fact may delay the diagnosis and treatment in many patients (12, 13).

Anal cancer treatment has evolvedsignificantly over the past three decades. Organ-preserving chemoradiation therapy (CRT) has been established asa standard of care for locally advanced diseaseinstead of abdominoperineal resection (14). This approach is formed of concurrent 5-fluorouracil (5-FU) or capecitabine and mitomycin C (MMC) with definitive radiation therapy. Chemoradiation offers a high level of disease control and successfully preserves the anal sphincter (15, 16).

Data on the outcomes of anal cancer from our region remains scarce. Herein, we report a series of patients treated at a tertiary cancer center, under the umbrella of a multidisciplinary clinic. We investigated patients and disease characteristics, and explored those who underwent non-oncologic resection before treatment.

## Materials and methods

### Patients’ population

We conducted a retrospective review of medical records for patients diagnosed with anal carcinoma and treated with definitive CRT at our institution from January 2007 to January 2020. Patients with metastatic disease, and those who received part of their CRT outside our center were excluded. Diagnosis was established through physical examinations, CT scan for the chest, abdomen, and pelvis, pelvic MRI, and colonoscopy with tumor biopsy. Pathology specimens from anal mass biopsies or hemorrhoidectomies were reviewed by a dedicated gastrointestinal pathologis.

### Chemoradiation

Management of all patients with locally advanced anal carcinoma were discussed at the institutional multidisciplinary board. Before 2013, radiation therapy utilized a conventional 3D conformal planning based on RTOG 9811 protocol, involving pelvic radiotherapy at 36 Gray (Gy) with a sequential boost to the primary disease and involved nodes up to 59 Gy (17). The introduction of Intensity Modulated Radiation Therapy (IMRT) marked a shift, with anal carcinoma patients being offered IMRT. The dose and target volume definition adhered to the RTOG 0529 protocol, utilizing dose-painted IMRT to mitigate grade 2 or more acute gastrointestinal and genitourinary toxicities. As per RTOG 0529, the dose to the primary tumor and lymph nodes was stage-dependent, and delivery was achieved using the simultaneous boost (SIB) dose painting technique. For T2N0 tumors, the primary PTV received a dose of 50.4 Gy over 28 fractions, with an elective nodal dose of 42 Gy. In the case of T3-T4 N0 tumors, the primary PTV was prescribed a dose of 54 Gy, while the nodal PTV was given 45 Gy over 30 fractions SIB (18).

In cases of node-positive disease, irrespective of the T stage, the administered dose wascontingent upon the size of the affected lymph nodes. Lymph nodes measuring 3 cm or less are prescribed 54 Gy for the tumor and 50.4 Gy for the nodal planning target volume (PTV) over 30 fractions using the simultaneous boost (SIB) technique. Conversely, when dealing with lymph nodes larger than 3 cm, a dose of 54 Gy was delivered to both the primary tumor and nodal PTVs over 30 fractions using the SIB approach (19).

Chemotherapy was administered using a regimen based on 5-FU/Capecitabine in combination with MMC or Cisplatin. The first regimen consisted of either 5-FU (1000 mg/m2/day) delivered continuously on days 1 to 4 and days 29 to 32, or Capecitabine (825 mg/m2 BID) on the days of radiotherapy, along with MMC (10 mg/m2) on Day 1 and Day 29. On the other hand, the second regimen involved the combination of 5-FU/Capecitabine with Cisplatin (75 mg/m2) on day 1 and day 29, maintaining the same doses for 5-FU or Capecitabine as in the previous protocol with MMC.

Assessment of response involved digital rectal examination, endoscopy, and pelvic MRI. Complete remission (CR) was characterized by the clinical and radiographic evidence confirming the complete disappearance of the tumor.

### Statistics

Statistical analyses were conducted using SPSS. The Chi-square test was employed to compare and identify differences between groups in terms of CR and recurrence rates. The primary endpoint, disease-free survival (DFS), was calculated from the date of the initial treatment to the date of death or recurrence (metastasis or local recurrence), while overall survival (OS) was measured from the date of the first treatment to the date of death due to anal cancer, with deaths from other causes considered as censored data. The Kaplan-Meier method was utilized to estimate 5-year DFS and 5-year OS for various groups, and differences in survival outcomes were evaluated using the log-rank test. A significance level of *p <*0.05 was applied to all analyses.

## Results

We identified records of 34 patients, 4 did not meet the inclusion criteria, leaving 30 patients for analysis. The mean age was 57 years (ranging from 32 to 80), 15 (50%) were males and 24 (80%) patients were above the age of 50 years. 21 (70%) were HPV positive, and the test failed in5 samples. **Table 1** illustrates patients and disease characteristics.

**Table 1:** Patients and disease characteristics.

Characteristics		Number (N)	Percentage(%)
**Age (years)**	≥50	24	80 %
	<50	6	20 %
**Gender**	Male	15	50 %
	Female	15	50 %
**Smoking**	Yes	18	60 %
	No	12	40 %
**Stage**	I	2	6.7 %
	II	13	43.3 %
	III	15	50 %
**Chemotherapy**	5 FU/Xeloda + MMC	23	76.7 %
	5 FU/Xeloda + Cisplatin	7	23.3 %
**HPV**	Positive	21	70 %
	Negative	4	13.3 %
	N/A	5	16.7 %
**Radiotherapy Technique**	3D	9	30 %
	IMRT	21	70 %
**Non- oncologic resection**	Yes	10	33.3 %
	No	20	66.7 %

24 (80%) patients attained CR following definitive CRT. The overall 5-year OS for the entire group was 78%. Notably, 20 out of 21 HPV-positive patients achieved CR, versus 1 out of 4 HPV-negative patients, *p*=0.006 3 out of 4 HPV negative patients experienced disease recurrence, compared to 3 out of 21 patients in the HPV-positive group, *p*= 0.03.

**Figure 1:**
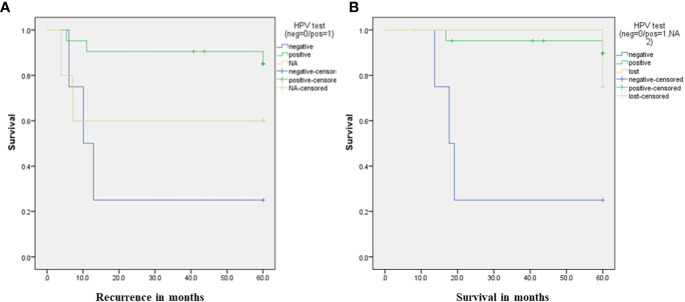
**(A)** Recurrence rate according to HPV status. **(B)** Survival rate according to HPV status.

The DFS for HPV-negative patients was 10 months,and not reached for HPV-positiv, *p*= 0.02. As regardOS, HPV-negative patients had a median OS of 17 months, whereas it was not reached for HPV-positive patients. The 5-year OS was 25% for HPV-negative patients compared to 89.6% for HPV-positive patients, *p*=0.001, as depicted in **Figure 1**.

10 patients underwent non-oncologic surgery upon diagnosis, with 9 (90%) of them achieving CR compared to 15 (75%) in the remaining 20 cases, *p*=0.51. The5-year OS for patients who underwent non-oncologic surgery was 73.6%, while for the other group was 88.9%, *p*=0.32.

**Figure 2:**
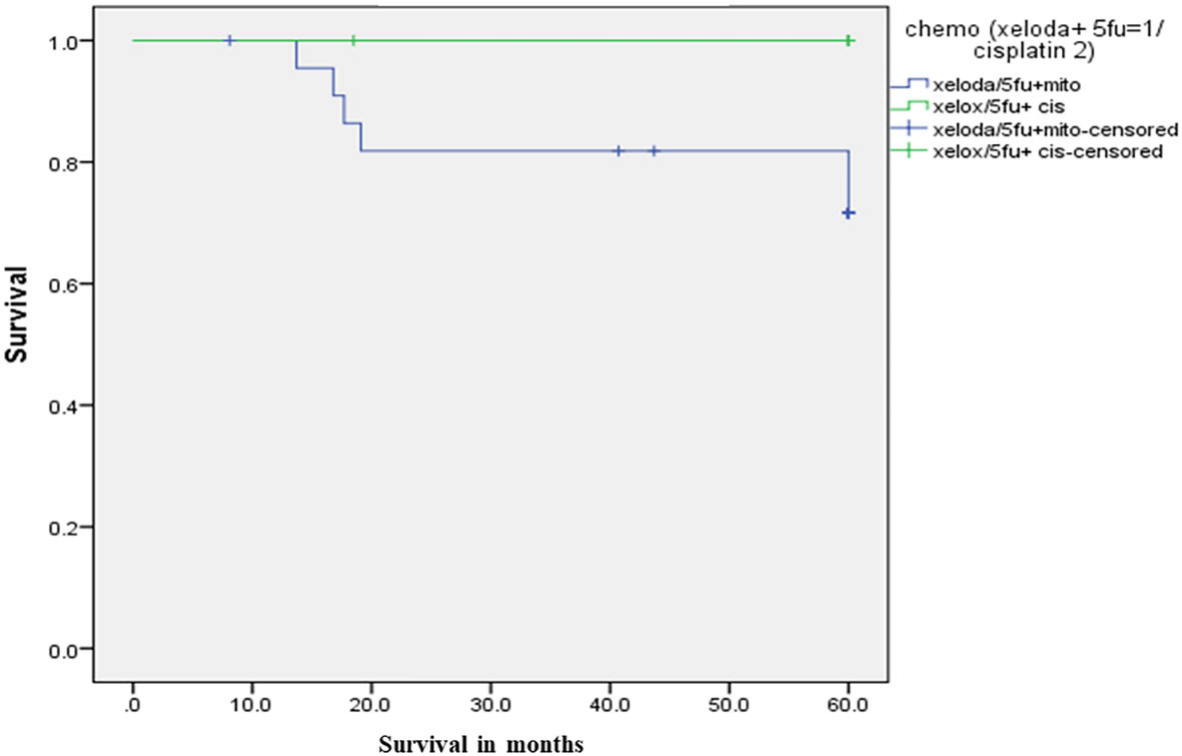
Survival curves according to the type of chemotherapy.

For chemotherapy, group 1 comprised 23 patients who received 5-FU/Capecitabine with MMC, 17 of them (73.9%) achieved CR. On the other hand, all the 7 patients in group 2, who received 5-FU/capecitabine with cisplatin, achieved CR, *p*=0.3. The 5-year DFS was 69% in group 1, compared to 85.7% in group 2, *p*=0.38. While 5-year OS was 71.6% in group 1 versus 100% in Group 2, *p*=0.14. as illustrated in **Figure 2**.

Regarding other patients and disease factors, **Table 2** demonstrated the potential impact on CR rate in addition to DFS and OS.

**Table 2:** Patient and disease factors in relation to disease outcomes.

Characteristics		pCR	*p* value	DFS	*p* value	OS	*p* value
**Age (years)**	≥50	79.2 %	0.34	75.0%	0.73	76.5 %	0.79
	<50	83.3 %		66.7%		83.3 %	
**Gender**	Male	86.7 %	0.54	80.0 %	0.38	85.7 %	0.33
	Female	73.3 %		66.7 %		70.6 %	
**Smoking**	Yes	73.7 %	0.43	68.0 %	0.66	77.4 %	0.067
	No	90.9 %		81.8 %		78.8 %	
**Stage**	I	100 %	0.83	100 %	0.26	100 %	0.76
	II	84.6%		84.6%		76.2%	
	III	73.3%		59.3%		77.1%	
**Chemotherapy**	5 FU/Xeloda + MMC	73.9%	0.3	69.0%	0.38	71.6%	0.14
	5 FU/Xeloda + Cisplatin	100.0%		85.7%		100.0%	
**HPV**	Positive	95.0%	0.006	85.0%	0.02	89.6%	0.001
	Negative	25.0%		25.0%		25.0%	
**Radiotherapy Technique**	3D	66.7 %	0.08	66.7 %	0.57	75.0 %	0.83
	IMRT	85.7 %		75.6 %		79.8 %	
**Non- oncologic resection**	Yes	90.0%	0.51	90.0%	0.15	89.9%	0.32
	No	75.0%		64.2%		73.6%	

With respect to treatment-related toxicity, skin reaction was the most common in 27 cases (90%) with grade 2 or higher, 5 patients (16.6%) experienced grade 2 diarrhea, and 1 patient (3.3%) developed a rectovaginal fistula.

## Discussion

In this retrospective analysis, we explored the clinical outcomes of patients with squamous cell carcinoma of the anal canal. To the best of our knowledge, this is the first report on anal cancer from the MENA region. We reported a 5-years OS of 78%, which aligns with survival rates reportedin the literature (5).

Although CRT is considered a standard treatment for locally advanced anal carcinoma surgery is typically reserved as a salvage option Nevertheless, our cohort demonstrated a distinctive situation, as one third of patients underwent non-oncologic surgery before being referred for chemoradiation. This fact may be attributed to the rarity of the disease and incidental detection of cancer in hemorrhoid specimens.

In large clinical trials, median age at diagnosis was typically above the age 50 years, 80% of patients in our cohort were diagnosed at age more than 50 years (20). Previous reports have indicated that diagnosis of anal SCC on top of hemorrhoids is not an isolated rare event, it was estimated that up to 4% of anal surgical specimens would uncover malignancy (11). Another trial had explored the risk of anal squamous cell cancer in patients with anal fistulas and fissures. They demonstrated increased risk of cancer in those patients even without presence of irritable bowel disease (21). However, these series did not explore the influence of non-oncologic resection on disease outcomes. In our study, we did not observe difference in oncologic outcomes in patient who underwent surgery before CRT. But we acknowledge that our study might be under-powered to detect the impact of non-oncologic resection.

Some data suggested that patients with HPV-negative tumors exhibited a reduced responsiveness to concurrent CRTand had a higher tendency for relapse (22). This finding came in concordance with our results, but this might be hampered withour small sample size and the fact that the majority of our patients tested positive for HPV.

In a systematic review, that tested the correlation between HPV status and treatment response (23), They found patients with HPV-positive status exhibited superior disease-free survival and overall survival compared to those with HPV-negative disease. This difference is attributed, at least in part, to the more favorable response to CRT in HPV-positive cases. Similarly, another study conducted in Japan, revealed a more favorable response in patients with HPV-positive anal cancer (24). The favorable response in HPV-positive may be related to distinct gene profiles in HPV-positive cancer cells. These differences are frequently identified among genes responsible for DNA regulation and repair, and cellular immune response. These distinctions enhance treatment sensitivity in these patients, especially the radiation effect (25). In our study, we demonstratedsuperior rates of CR, DFS, and OS among patients with HPV-positive status compared with HPV-negative status. Aforementioned, the small number of HPV-negative cases might affect the robustness of our results.

In our study, we observed no statistical difference in outcomes based on the type of chemotherapy. A randomized controlled trial by Ajani et al, compared recurrence rates between MMC-5FU and cisplatin5FU, the recurrence rate with MMC was 25% and 33% in the cisplatin group, but the difference between the groups was not statistically significant, (16). In our series, we found comparable results, with a 30.4% local disease recurrence in the MMC group compared to 14.3% in the cisplatin group.

In the ACT-II trial, (26) which assessed the complete response rate between the MMC and cisplatin groups with radiation therapy, including or excluding maintenance treatment, there was no significant difference between the two groups. The complete response rates were 89.6% in the cisplatin arm and 90.5% in the MMC arm, *p*=0.64. Noteworthy, there was nomaintenance chemotherapy after CRT in our cohort.

A study by O’Brien and colleagues compared patients with hemorrhoidal SCC to those with non-hemorrhoidal SCC. They reported a higher proportion of stage I/II in the hemorrhoidal SCC arm, but no significant difference in OSbetween the two groups (27). These findings aligned with our data, indicating that hemorrhoidal surgery did not impact the oncological outcomes of the underlying anal SCC. Furthermore, It is important to recognize that local excision alone is acceptable treatment for early-stage anal cancer (28).

The emergence of immunotherapy in the treatment of anal cancers is promising. Recent studies have demonstrated encouraging results, and further trials in this context are anticipated (29).

We acknowledge that our cohort has multiple limitations, first is the retrospective nature of the study and potential selection bias. Second is the number of patients in our series is relatively small, which restricts the generalizability of the results and reduces statistical power. Third, is the differences in the treatment approach among the patient’s groups, which might have influence the outcomes. Nevertheless, this investigation offers the initial set of oncological outcomes for anal cancer in our region. It aims to enhance comprehension of this underreported disease.

## Conclusion

This report evaluated the oncological outcomes of patients diagnosed with locally advanced anal carcinoma undergoing CRT. Our findings reveal comparable oncological outcomes to those reported in the literature for patients with anal cancer. Notably, we observed significantly higher rates of CR, DFS, and OS in HPV-positive patients compared to their HPV-negative counterparts. Noteworthy, non-oncologic resection before CRT did not seem to impact oncologic outcomes. Future prospective trials are essential to validate our results and further enhance our understanding of anal cancer.

## Data availability statement

The original contributions presented in the study are included in the article/supplementary material. Further inquiries can be directed to the corresponding author.

## Author contributions

TA: Conceptualization, Formal analysis, Supervision, Writing – original draft, Writing – review & editing. RA: Supervision, Writing – original draft, Writing – review & editing, Methodology. IM: Methodology, Writing – review & editing. SA (4th author): Investigation, Writing – original draft. HA: Data curation, Formal analysis, Writing – original draft. RT: Data curation, Investigation, Writing – review & editing. SA (7th author): Conceptualization, Methodology, Writing – original draft. YK: Methodology, Writing – review & editing. FA: Methodology, Writing – original draft, Writing – review & editing.
